# Supplementary material for: Recursive self-embedded vocal motifs in wild orangutans
Source: eLife. 2024 Jan 22;12:RP88348. doi: 10.7554/eLife.88348 (PMC10945596; doi:10.7554/eLife.88348)
Supplement: Supplementary file 1. [file elife-88348-supp1.docx]

**Supplementary Materials**

**Count of obs ~ Pulse level *Type + (1|ID Contribution)**

Pulse level: BSP, FP, GSP, SBTE

Type: ON or OFF isochrony

| **Model** | **Df** | **AIC** | **BIC** | **logLik** | **deviance** | **Chisq** | **ChiDf** | **Pr(>Chisq)** |
| --- | --- | --- | --- | --- | --- | --- | --- | --- |
| ***null*** | 4 | 2398.896 | 2414.092 | -1195.448 | 2390.896 | - | - | - |
| ***full*** | 11 | 2114.608 | 2156.398 | -1046.304 | 2092.608 | 298.288 | 7 | p<0.001 |

| **Random effects** |  |  |  |  |  |
| --- | --- | --- | --- | --- | --- |
| Conditional model |  |  |  |  |  |
| Groups | Name | Variance | Std.Dev. |  |  |
| ID contribution | (Intercept) | 0.2705 | 0.5201 |  |  |
| Number of obs: 330. groups: ID contribution, 65 | |  |  |  |  |
|  |  |  |  |  |  |
| **Conditional model:** |  |  |  |  |  |
|  | **Estimate** | **Std Error** | **zvalue** | **Pr(>\|z\|)** |  |
| (Intercept) |  |  |  |  |  |
| Type (ON) ^a^ | 1.2959 | 0.1445 | 8.97 | <0.0001 | *** |
| Pulse level (FP)^a^ | -1.3532 | 0.1670 | -8.10 | <0.001 | *** |
| Pulse level (GSP) ^a^ | -0.8826 | 0.2449 | -3.60 | <0.001 | *** |
| Pulse level (SBTE) ^a^ | -2.3268 | 0.2966 | -7.85 | <0.001 | *** |
| Pulse level (FP) : type (ON) ^a^ | 1.0314 | 0.2150 | 4.80 | <0.001 | *** |
| Pulse level (GSP) : type (ON) ^a^ | 0.1615 | 0.3241 | 0.50 | 0.618283 |  |
| Pulse level (SBTE) : type (ON)^a^ | -0.1903 | 0.3626 | -0.52 | 0.599660 |  |

*^a^Pulse level (BSP) and Type (OFF) being the reference categories*

| ***Post-hoc* test** | | | | | |
| --- | --- | --- | --- | --- | --- |
| **Contrast** | **Estimate** | **SE** | **df** | **t.ratio** | **p.value** |
| FP: OFF - ON | -2.57 | 0.161 | 319 | -15.957 | <.0001 |
| BSP: OFF - ON | -1.54 | 0.144 | 319 | -10.640 | <.0001 |
| GSP: OFF - ON | -1.70 | 0.286 | 319 | -5.940 | <.0001 |
| SBTE: OFF - ON | -1.35 | 0.333 | 319 | -4.048 | 0.0001 |
